# Systematic review and meta-analysis of third-line salvage therapy for the treatment of advanced non-small-cell lung cancer: A meta-analysis of randomized controlled trials

**DOI:** 10.18632/oncotarget.24967

**Published:** 2018-03-23

**Authors:** Nan Zhang, Nan Guo, Liang Tian, Zhigang Miao

**Affiliations:** ^1^ Department of Thoracic Surgery, Cangzhou Central Hospital, Cangzhou, Hebei Province, China; ^2^ Department of Cardiology, Cangzhou Central Hospital, Cangzhou, Hebei Province, China; ^3^ Department of Pathology, Cangzhou Central Hospital, Cangzhou, Hebei Province, China

**Keywords:** non-small-cell lung cancer, third-line therapy, heavily pretreated, meta-analysis

## Abstract

**Purpose:**

We performed a systematic review and meta-analysis to investigate the efficacy of third-line treatment for advanced non-small-cell lung cancer (NSCLC).

**Materials and Methods:**

Relevant trials were identified by searching electronic databases and conference meetings. Prospective randomized controlled trials (RCTs) assessing third-line therapy in advanced NSCLC patients were included. Outcomes of interest included overall survival (OS) and progression-free survival (PFS).

**Results:**

A total of 1,985 advanced NSCLC patients received third-line treatment from 11 RCTs were included for analysis. The use of single targeted agent as third-line therapy for advanced NSCLC did not significantly improved PFS (HR 0.75, 95% CI: 0.28–2.04, *p* = 0.58) and OS (HR 1.01, 95% CI: 0.86–1.17, *p* = 0.95) when compared to docetaxel alone. In addition, erlotinib-based doublet combination therapy did not significantly improved PFS (HR 0.94, 95% CI: 0.78–1.13, *p* = 0.49) and OS (HR 1.08, 95% CI: 0.78–1.51, *p* = 0.65) in comparison with erlotinib alone.

**Conclusions:**

The findings of this study show that the efficacy of single novel targeted agent is comparable to that of docetaxel alone in terms of PFS and OS for heavily pretreated NSCLC patients. In addition, no survival benefits are obtained from erlotinib-based doublet therapy, thus single agent erlotinib could be recommended as third-line treatment for unselected advanced NSCLC patients.

## INTRODUCTION

Despite a significant improvement in diagnostics and therapy during the past decade, lung cancer remains the leading cancer-related deaths around the world [1]. The majority of lung cancer cases (approximately 80–85%) are classified as non-small-cell lung cancer (NSCLC), including squamous carcinoma, adenocarcinoma and large cell carcinoma [2]. Only 30% of NSCLC patients are resectable at the initial diagnosis, while the remaining 70% of NSCLC have metastatic or locally advanced disease at time of diagnosis. Palliative chemotherapy and/or radiotherapy represent the standard of care for these patients. Until now, platinum based doublets with third generation agents remains the standard of first line advanced NSCLC treatment [3–5]. However, most patients receiving front-line chemotherapy would eventually become refractory to chemotherapy or experience disease progression after a certain period of time. Currently, two cytotoxic agent docetaxel and pemetrexed and the biologic agent erlotinib have been approved as second-line treatment for advanced NSCLC patients [3, 6]. Two previously published trials demonstrate that docetaxel is superior to best support care (BSC), vinorelbine, or ifosfamide, in terms of survival benefits and quality of life (QoL) for the treatment of advanced NSCLC patients previously treated with platinum-based chemotherapy [7, 8]. In another large non-superiority phase III trials, pemetrexed is compared with docetaxel in patients with good PS (0–2), and the result shows no significant difference in overall survival between pemetrexed and docetaxel (8.3 months vs 7.9 months, HR, 0.99; *P* = 0.226), but with less toxicities of pemetrexed [9]. Erlotinib, the third available option, has proven superior to BSC, significantly improving overall survival (6.7 months versus 4.7 months, HR = 0.70; *P* < 0.001) and progression-free survival (2.2 months versus 1.8 months, HR = 0.61; *p* < 0.001) [10]. However, standard therapeutic options beyond second-line treatment are insufficient. To our best knowledge, these is no prospective trials specifically addressing the role of third-line treatment in advanced NSCLC, thus we conduct this meta-analysis of randomized controlled trials reporting survival data of those patients who have already received ≥ 2 prior regimens to clearly determine the role of third-line treatment in NSCLC.

## MATERIALS AND METHODS

### Study design

We performed this systematic review and meta-analysis according to the Reporting Items for Systematic Reviews and Meta-Analyses (PRISMA) statement guidelines 2009 [11].

### Search strategy

We conducted a comprehensive literature search of public databases including PubMed, EMBASE, and the Cochrane library (up to May 30, 2017). Relevant search keywords including the followings: ‘‘non-small-cell lung cancer,’’ ‘‘third-line therapy,’’ “pretreated” and ‘‘randomized controlled trials.’’ No language restriction was administered. We also conducted a manual search of conference proceedings. All results were input into Endnote X7 reference software (Thomson Reuters, Stamford, CT, US) for duplication exclusion and further reference management (Supplementary Table 1 search strategy for EMBASE).

### Study selection

Clinical trials that met the following criteria were included: (1) prospective phase II or III trials involving NSCLC patients; (2) patients received second or later-line therapy; and (3) available survival data regarding third-line treatment in advanced NSCLC patients. If multiple publications of the same trial were retrieved or if there was a case mix between publications, only the most recent publication (and the most informative) was included.

### Data extraction

Two independent investigators conducted the data abstraction, and any discrepancy between the reviewers was resolved by consensus. The following information was extracted for each study: first author’s name, year of publication, trial phase, number of enrolled subjects, treatment arms, median age, median progression-free survival, and overall survival.

### Outcome measures

A formal meta-analysis was conducted using Comprehensive Meta Analysis software (Version 2.0). The outcome data were pooled and reported as hazard ratio (HR). The primary outcome of interest was OS and secondary outcomes PFS in advanced NSCLC receiving third-line therapy.

### Statistical analysis

All statistical analyses were performed by using Version 2 of the Comprehensive MetaAnalysis program (Biostat, Englewood, NJ). Between-study heterogeneity was estimated using the χ^2^-based *Q* statistic [12]. The *I*^2^ statistic was also calculated to evaluate the extent of variability attributable to statistical heterogeneity between trials. A statistical test with a *p*-value less than 0.05 was considered significant. Study quality was assessed by using the Jadad scale based on the reporting of the studies’ methods and results [13].

To assess the potential risk bias of included trials, we used the Cochrane risk of bias tool, which had seven domains including random sequence generation, allocation concealment, blinding of participants and personnel, blinding of outcome assessment, incomplete outcome data, selective reporting and other bias. The classification of the judgment for each domain was low risk of bias, high risk of bias, or unclear risk of bias and two authors independently evaluated the risk of studies.

## RESULTS

### Search results

**Figure 1:**
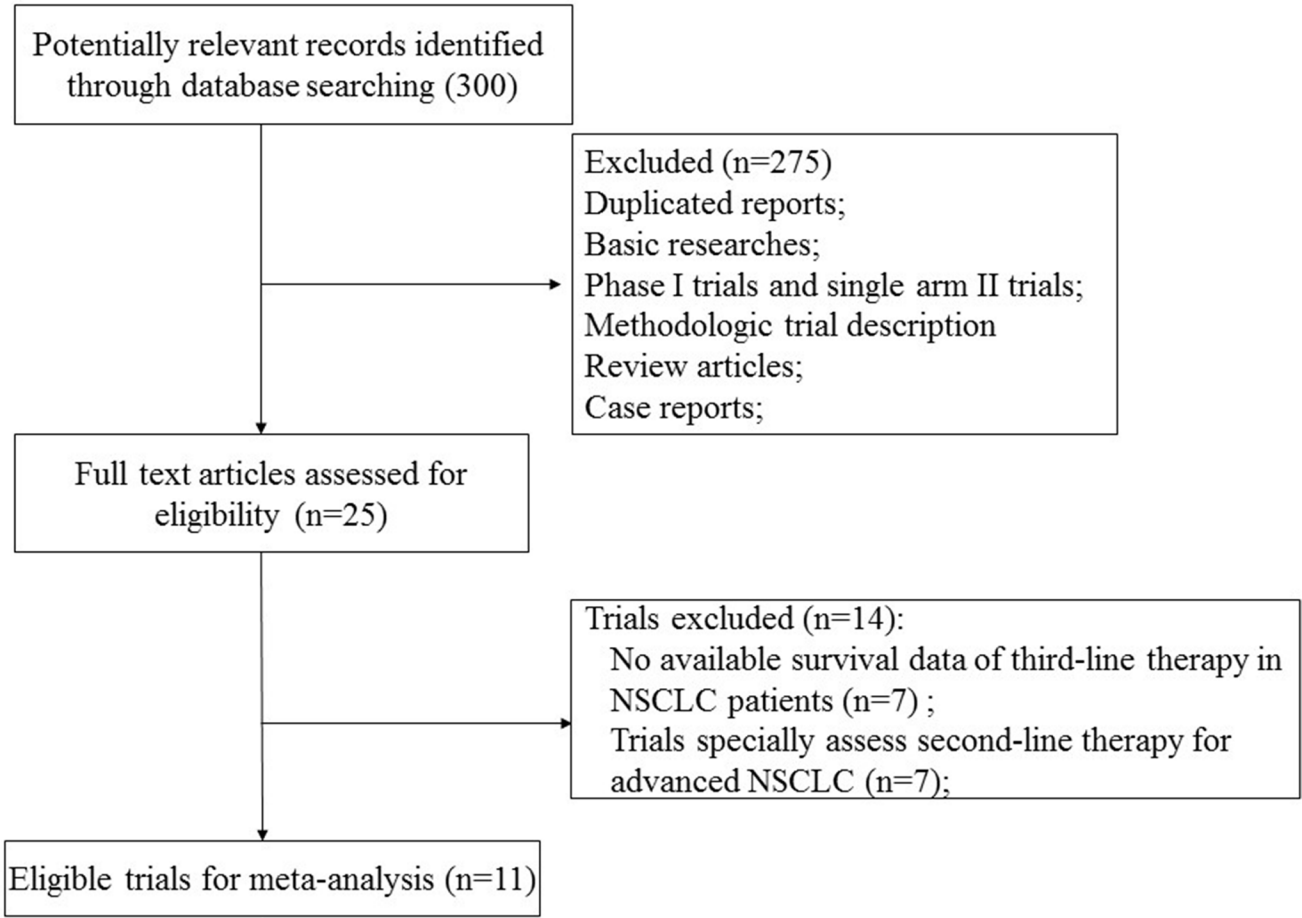
Studies eligible for inclusion in the meta-analysis

We initially found 300 relevant citations of treatment therapy in pre-treated NSCLC patients. After excluding review articles, phase I studies, case reports, editorial, letters, commentaries, meta-analyses and systematic review (Figure 1), we retrieved 25 reports for full-text screening. Seven of included trials were not used because these studies only included patients receiving second-line therapy [14–20]. Another seven trials were excluded because these studies did not report survival data of patients received third-line therapy [20–26]. Finally, we selected 11 randomized controlled trials for analysis in the present study [10, 26–35]. Five randomized trials compared erlotinib-based doublet versus erlotinib as third-line therapy in advanced NSCLC [29–31, 33, 35], while the remaining trials investigated single targeted agent versus docetaxel/placebo as third-line therapy for advanced NSCLC. A total of 1.958 patients received third-line therapy were included for analysis. Table 1 listed the baseline characteristics of patients and studies. The quality of each included study was roughly assessed according to Jadad scale, and six of the eleven randomized controlled trials were double-blind placebo-controlled trials, thus had Jadad score of 5. Another seven trials were an open-label controlled trials, thus had Jadad score of 3.

**Table 1:** Baseline characteristics of 11 included trials

Study/year	phase	No. of elderly patients	treatment regimen	median age in all treatment cohorts	primary endpoint	Jadad Score
**Shepherd F.A. et al/2005**	III	362	erlotinib 150 mg qd po	62	OS	5
			placebo	59		
**Kim E.S. et al/2008**	III	226	gefitinib 250 mg qd po	61	OS	3
			docetaxel 75 mg/m^2^	60		
**Maruyama R. et al/2008**	III	75	gefitinib 250 mg qd po	NR	OS	3
			docetaxel 60 mg/m^2^	NR		
**Sequist L.V. et al/2011**	II	34	Tivantinib +erlotinib 150 mg qd po	64	PFS	5
			Placebo+erlotinib 150 mg qd po	62		
**Miller V.A. et al/2012**	IIb/III	159	afatinib 50 mg qd po	58	OS	5
			placebo	59		
**Scagliotti G.V. et al/2012**	III	269	Sunitinib +erlotinib 150 mg qd po	61	OS	5
			Placebo+erlotinib 150 mg qd po	61		
**Spigel D.R. et al/2013**	II	22	Onartuzumab +erlotinib 150 mg qd po	63	PFS	5
			Placebo +erlotinib 150 mg qd po	64		
**Borghaei H. et al/2015**	III	66	Nivolumab 3 mg/kg q.2.w.	61	OS	3
			docetaxel 75 mg/m^2^	64		
**Scagliotti G. et al/2015**	III	354	Tivantinib +erlotinib 150 mg qd po	62	OS	5
			Placebo +erlotinib 150 mg qd po	61		
**Rittmeyer A. et al/2017**	III	210	Atezolizumab 1200 mg	63	OS	3
			docetaxel 75 mg/m^2^	64		
**Spigel D.R. et al/2017**	III	181	Onartuzumab +erlotinib 150 mg qd po	62	OS	5
			Placebo +erlotinib 150 mg qd po	63		

Abbreviations: OS, overall survival; PFS, progression-free survival; NR, not reported.

### Risk of bias in included studies

Supplementary Figure 1 and Supplementary Figure 2 showed risk bias in all 11 studies. All of the included studies (100%) described random sequence generation. five studies (45%) described adequate allocation concealment. Seven studies (63.6%) described blinding of participants and personnel. Four studies had high risk of bias about blinding of participants and personnel because these four studies were open label trial. Nine studies had a low risk of incomplete outcome data. Although some researches had dropout, the effect of intervention was not affected due to due to the small scale of dropout. Ten studies had low risk of selectively reporting results.

### Single agent therapy as third-line therapy

**Figure 2:**
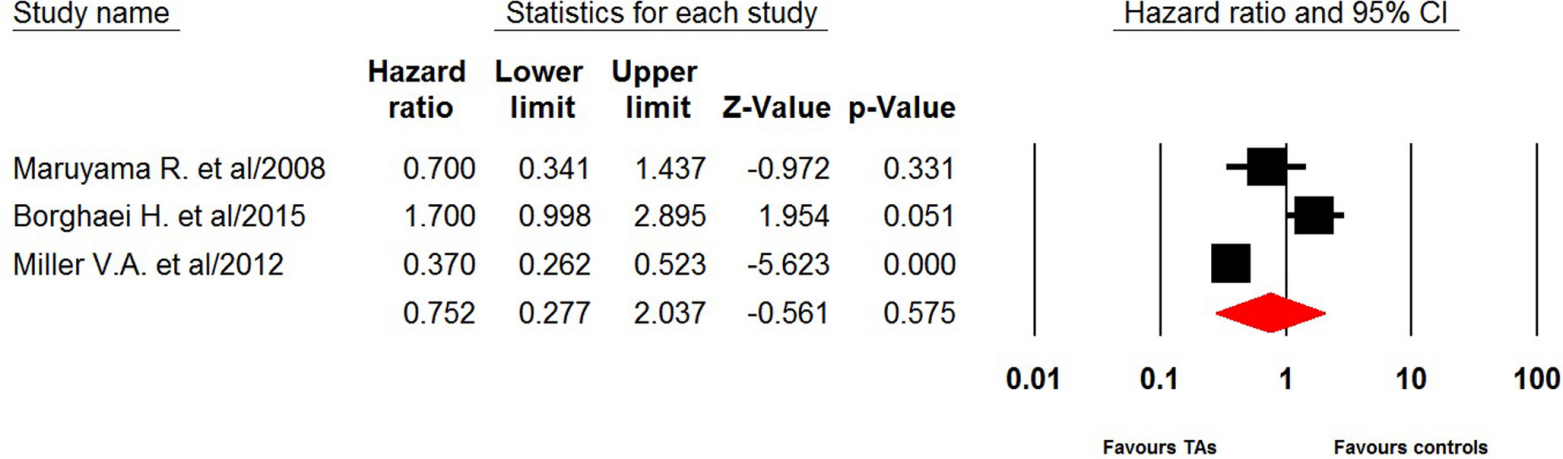
Random-effect model of hazard ratio (95%CI) of PFS associated with single targeted agent versus placebo/docetaxel in NSCLC patients

**Figure 3:**
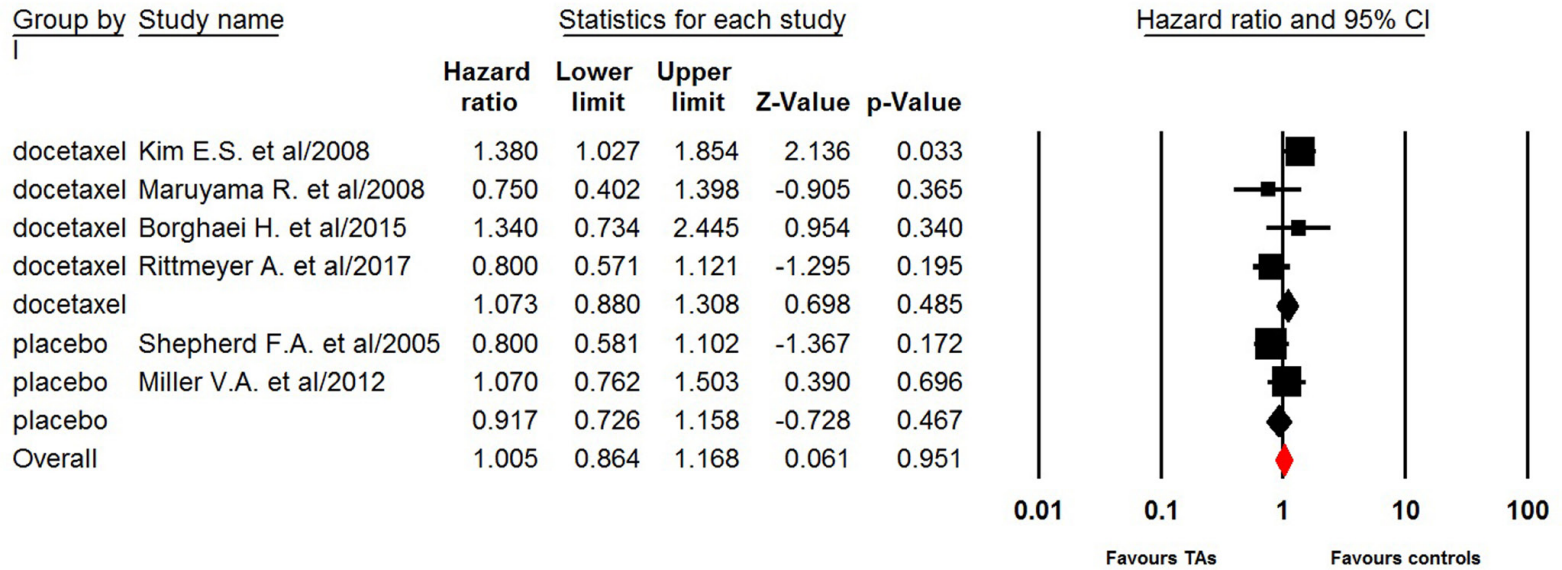
Fixed-effect model of hazard ratio (95%CI) of OS associated with single targeted agent versus placebo/docetaxel in NSCLC patients

Three trials reported PFS data of single agent third therapy in NSCLC patients. The pooled hazard ratio for PFS demonstrated that the single agent third therapy in advanced NSCLC patients did not significantly improved PFS giving HR 0.75 (95% CI: 0.28–2.04, *p =* 0.58, Figure 2), in comparison with docetaxel/placebo. There was significant heterogeneity between trials (*I*^2^ = 92.0%, *p* < 0.001), and the pooled HR for PFS was performed by using random-effects model. Six trials reported OS data of single targeted agent as third-line therapy in this patient population. The pooled hazard ratio for OS showed that the use of single targeted agent as third therapy did not significantly improved OS giving HR 1.01 (95% CI: 0.86–1.17, *p =* 0.95, Figure 3), in comparison with docetaxel/placebo. Sub-group analysis according to controlled therapy showed that the use of single targeted agent as third therapy did not significantly improved OS in comparison with docetaxel (HR 1.07, 95% CI: 0.88–1.31, *p* = 0.49) or placebo (HR 0.92, 95%CI: 0.73–1.16, *p* = 0.47).

### Erlotinib-based combination as third therapy

**Figure 4:**
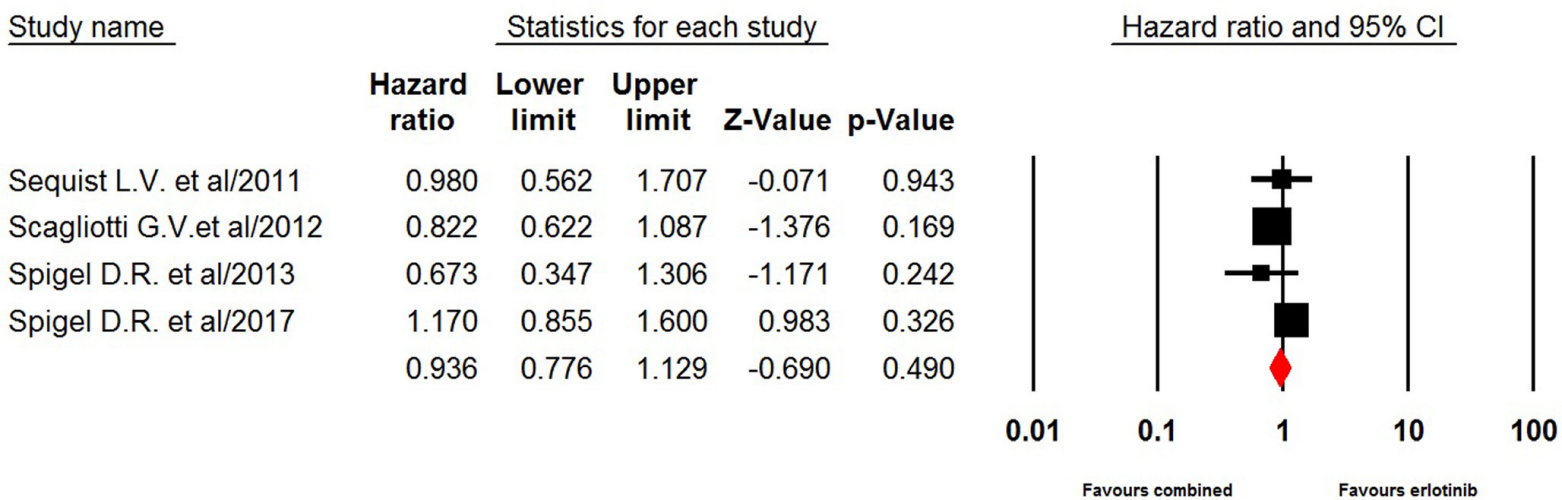
Fixed-effects model of hazard ratio (95%CI) of PFS associated with erlotinib-based doublet versus erlotinib in NSCLC patients

**Figure 5:**
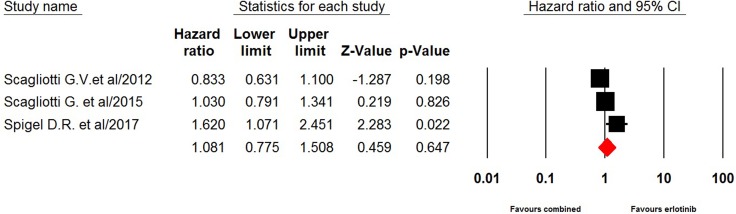
Fixed-effects model of hazard ratio (95% CI) of OS associated with erlotinib-based doublet versus erlotinib in NSCLC patients

Four included trials comparing erlotinib-based doublet versus erlotinib alone as third-line therapy reported survival data. The pooled hazard ratio for PFS demonstrated that erlotinib-based doublet combination therapy in heavily treated NSCLC patients did not significantly improved PFS (0.94, 95% CI: 0.78–1.13, *p* = 0.49, Figure 4) and OS (HR 1.08, 95% CI: 0.78–1.51, *p* = 0.65, Figure 5) when compared to erlotinib alone.

### Publication bias

We did not perform publication bias analysis due to limited randomized controlled trials in the present study.

## DISCUSSION

Until now, platinum-based doublet chemotherapy represents the gold standard in the treatment of chemotherapy-naïve advanced NSCLC [36]. Unfortunately, most of NSCLC patients ultimately suffer from disease progression. In the past few years, the second-line treatment options for advanced NSCLC have been established. Two chemotherapeutic agents, docetaxel and pemetrexed, and the biological drug erlotinib are the only three drugs approved for clinical use in this setting, achieving a median 8–10 months of overall survival [6, 37]. However, clinicians inevitably encounter difficulty in treating patients with advanced NSCLC who experience a relapse following second-line treatment with these drugs, and it has been reported that more than 38% of advanced NSCLC patients who received first-line chemotherapy could receive third-line chemotherapy [38]. In addition, this patient population has been increasing, and there is an urgent need to clearly define the role of third-line treatment for advanced NSCLC [39, 40]. To our best knowledge, there is no prospective randomized controlled trial specifically assessed the role of third-line treatment in advanced NSCLC patients, we thus conduct this meta-analysis of randomized controlled trials with predefined sub-group analysis to determine the role of third-line treatment for this patient population.

A total of 1,985 advanced NSCLC patients received third-line treatment from 11 RCTs are included for analysis. The quality of included trials is high. Seven of the eleven trials are double-blinded, placebo-controlled trials and the other four trials are open-label randomized controlled trials. The pooled results show that single targeted agent as third-line therapy for advanced NSCLC does not significantly improve PFS (HR 0.75, 95% CI: 0.28–2.04, *p* = 0.58) and OS (HR 1.01, 95% CI: 0.86–1.17, *p* = 0.95) when compared to placebo/docetaxel alone. In the setting of the third-line therapy, single agent docetaxel is one of the most frequently used regimens for heavily pretreated NSCLC patients. Several retrospective studies have demonstrated that advanced NSCLC patients with good performance status might benefit from cytotoxic chemotherapy including docetaxel alone [41–44], but these findings need to be confirmed in prospective randomized trials. As a result, the American Society of Clinical Oncology (ASCO) clinical practice guideline could not make a recommendation for or against using cytotoxic agents as third-line therapy [6]. In consistent with previous findings, sub-group analysis according to controlled therapy shows that the use of single targeted agent as third therapy does not significantly improve OS in comparison with docetaxel alone (HR 1.07, 95% CI: 0.88–1.31, *p* = 0.49). As a result, prospective randomized controlled specially comparing novel targeted agent with docetaxel as third-line therapy for advanced NSCLC are clearly needed.

Until now, erlotinib is the only recommended third-line therapy for patients who have not received prior erlotinib or gefitinib according to ASCO clinical practice guideline [3]. In the present study, four included trials investigate whether the addition of a novel target to erlotinib would improve survival in heavily pretreated NSCLC patients. The pooled results show that no obvious benefits are obtained from combination therapy in terms of PFS (0.94, *p* = 0.49) and OS (HR 1.08, *p* = 0.65) when compared to erlotinib alone. Based on our findings, single agent erlotinib remains the recommended third-line therapy for advanced NSCLC patients who not received prior erlotinib or gefitinib.

There are several limitations exist in this analysis. First, this meta-analysis only includes published trials, and a meta-analysis of individual level data might define more clearly treatment benefits in specific subgroups. For instance, anti-EGFR TKIs have been shown to have excellent treatment outcome in patients with EGFR mutation, and we are unable to investigate whether the survival benefit from third-line erlotinib is similar in NSCLC patients with or without EGFR mutation. Second, none of the included trials report the toxicities of third-line therapy in heavily pretreated NSCLC patients. Thus, we could not answer whether the use of erlotinib-based doublet combination therapy in this patient population would increase the toxicities in comparison with erlotinib alone. Third, different targeted agents, including EGFR-TKIs and immune check point inhibitors, are included for analysis in the present study, which might increase the heterogeneity among included trials. Fourthly, our analyses are based on subgroup data from individual trials and thus lack power. Also, none of the combination treatments examined in the meta-analysis are licensed. Finally, publication bias is an important issue for meta-analysis because trials with positive results are more likely to be published. Our paper do not assess publication bias due to limited trials included for analysis.

## CONCLUSIONS

In conclusion, this is the first-meta-analysis specifically assessing the efficacy of third-line therapy in the treatment of advanced NSCLC patients. The results of our study suggest that the efficacy of single novel targeted agent is comparable to that of docetaxel alone in terms of PFS and OS for heavily pretreated NSCLC patients. In addition, no survival benefits are obtained from erlotinib-based doublet therapy, thus single agent erlotinib could be recommended as third-line treatment for unselected advanced NSCLC patients. Further studies are recommended to specifically investigate the efficacy and toxicities of third-line therapy in the treatment of advanced NSCLC patients.

## SUPPLEMENTARY MATERIALS FIGURES AND TABLE


