# Supplementary material for: Systematic review and meta-analysis of third-line salvage therapy for the treatment of advanced non-small-cell lung cancer: A meta-analysis of randomized controlled trials
Source: Oncotarget. 2018 Mar 23;9(83):35439–47. doi: 10.18632/oncotarget.24967 (PMC6226041; doi:10.18632/oncotarget.24967)
Supplement: Supplementary file 1 [file oncotarget-09-35439-s001.pdf]

Systematic review and meta-analysis of third-line salvage therapy for the treatment of advanced non-small-cell lung cancer: A meta-analysis of randomized controlled trials

SUPPLEMENTARY MATERIALS

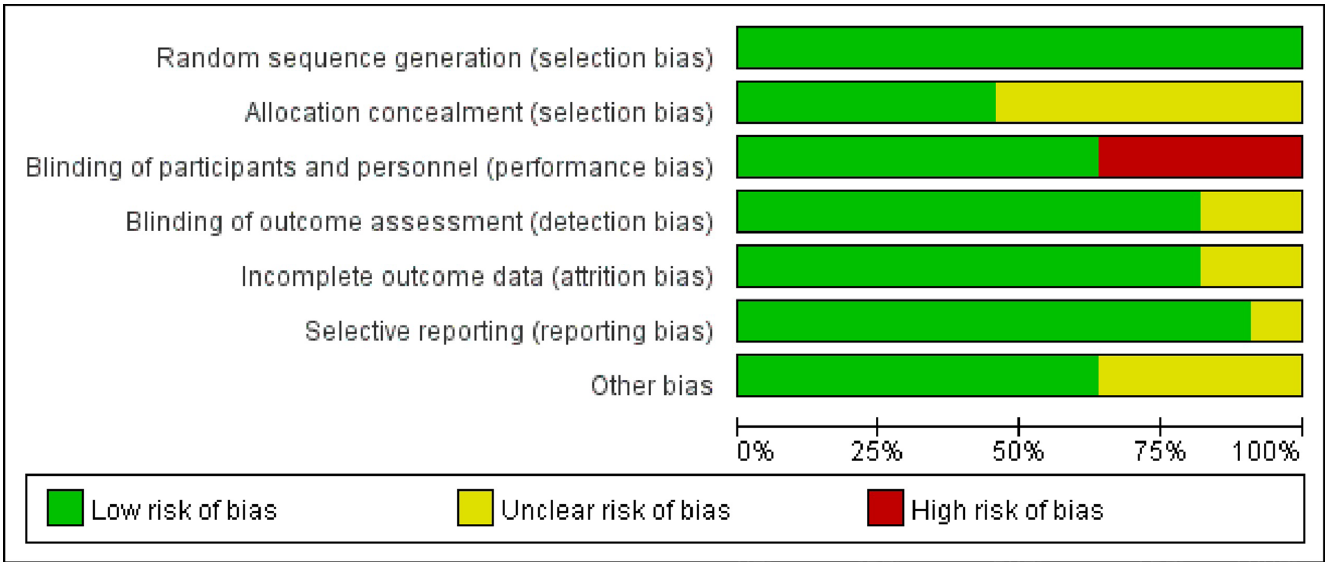

Supplementary Figure 1: Risk of bias graph: review authors' judgements about each risk of bias item presented as percentages across all included studies.

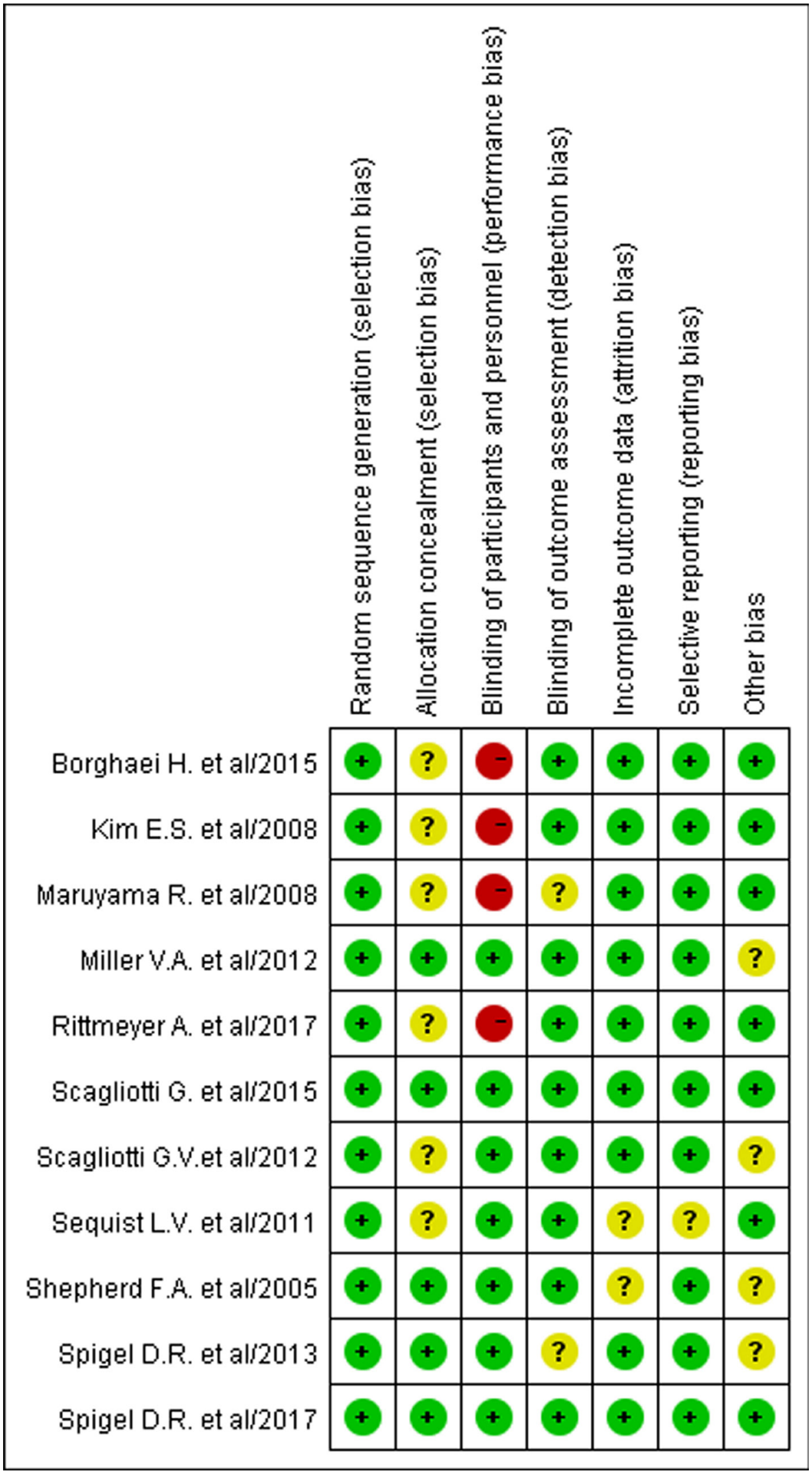

Supplementary Figure 2: Risk of bias summary: review authors' judgements about each risk of bias item for each included study.

Supplementary Table 1: EMBASE search strategy

#1 'Clinical trial'/
#2 'Randomized controlled trial'/
#3 Randomization/
#4 Single-Blind Method/
#5 Double-Blind Method/
#6 Cross-Over Studies/
#7 'Random Allocation'/
#8 Placebo/
#9 Randomized controlled trial*.tw.
#10 Rct.tw.
#11 Random allocation.tw.
#12 Randomly allocated.tw.
#13 Allocated randomly.tw.
#14 (allocated adj2 random).tw.
#15 Single blind*.tw.
#16 Double blind*.tw.
#17 ((treble OR triple) adj blind*).tw.
#18 Placebo*.tw.
#19 Prospective study/
#20 OR/#1-#19
#21 Case study/
#22 Case report.tw.
#23 Abstract report/ OR letter/
#24 OR/#21-#23
#25 #20 NOT #24
#26 (Lung* OR Respiratory* OR Bronchial*).mp. [mp=title, abstract, subject headings, heading word, drug trade name, original title, device manufacturer, drug manufacturer name]
#27 (carcin* OR cancer* OR neoplasm* OR tumour* OR tumor* OR cyst* OR adenocarcin* OR malign*).mp.
#28 exp Squamous/
#29 OR/#26-#28
#30 exp Adenocarcinoma/ Bronchiole alveolar carcinoma/ Large cell undifferentiated carcinoma
#31 #27 AND (#29 OR #30)
#32 metastatic/ OR advanced/
#33 previously treated/ OR second-line/ OR third-line
#34 #32 OR #33
#35 #25 AND #31 and #34
